# The scarcity-weighted water footprint provides unreliable water sustainability scoring

**DOI:** 10.1016/j.scitotenv.2020.143992

**Published:** 2021-02-20

**Authors:** Davy Vanham, Mesfin M. Mekonnen

**Affiliations:** a European Commission, Joint Research Centre (JRC), Ispra, Italy; b Department of Civil, Construction and Environmental Engineering, University of Alabama, Tuscaloosa, AL, United States

**Keywords:** LCA, Water use, Water stress, SDG6, Water efficiency, Water productivity

## Abstract

To evaluate the environmental sustainability of blue water use or the blue water footprint (WF) of a product, organisation, geographical entity or a diet, two well-established indicators are generally applied: water efficiency and blue water stress. In recent years, the Life Cycle Assessment (LCA) community has developed, used and promoted the indicator scarcity-weighted WF, which aims to grasp both blue water use and blue water stress in one indicator. This indicator is now recommended in an ISO document on water footprinting and many scholars have used associated scarcity-weighted water use indicators. However, questions on its physical meaning and its ability to correctly evaluate water sustainability have emerged. Here, we analyse for global irrigated wheat production to what extend the scarcity-weighted WF addresses blue water stress and water efficiency. We observe inconsistent results, as a significant proportion of unsustainably produced irrigated wheat has better scarcity-weighted WF scores as compared to sustainably produced irrigated wheat. Using the scarcity-weighted WF or scarcity-weighted water use for policy-making including product labelling, punishes some farmers producing their wheat in a water-sustainable way and promotes some farmers producing wheat unsustainably. Applying the scarcity-weighted WF indicator thereby is contraproductive in reaching the Sustainable Development Goal (SDG) target 6.4 on reducing water stress. In line with the specifications of this SDG target, to evaluate the sustainability of blue water use or the blue WF, the two indicators water stress and water efficiency should be used separately, in a complementary way.

## Highlights

• We test the scarcity-weighted water footprint (S—W WF) on its physical meaning.
• We use the established indicators blue water stress and water efficiency to do so.
• For global irrigated wheat production the S—W WF provides unreliable scoring.
• Unsustainably produced wheat is better ranked than sustainably produced wheat.
• Using the S—W WF for decision-making is contraproductive for achieving SDG target 6.4.

## Graphical abstract

**Figure:**
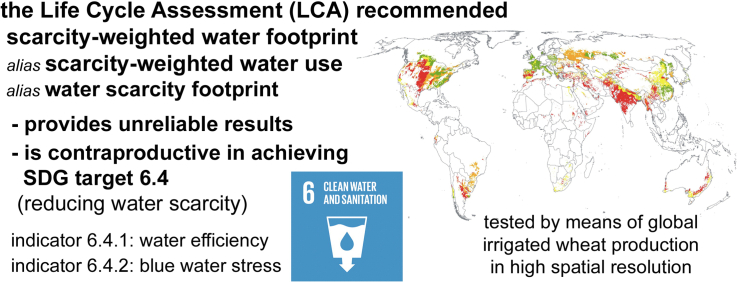

## 1 Introduction

The water footprint (WF) has been introduced in 2002 (Hoekstra and Hung, 2002), as an indicator of direct and indirect water use by a producer or consumer, showing how water flows through our economies by tracing it through supply chains and trade. It quantifies blue and green water consumption (Hoekstra and Mekonnen, 2012), as both are important for food production (Falkenmark et al., 2019; Rockström et al., 2009; Rockström et al., 2007), energy production (Mekonnen et al., 2015) and the environment (Falkenmark et al., 2019; Schyns et al., 2019; Vanham et al., 2018). Blue water refers to liquid water in rivers, lakes, wetlands and aquifers. Green water is the soil water held in the unsaturated zone, formed by precipitation and available to plants. Irrigated agriculture receives blue water (from irrigation) as well as green water (from precipitation), while rainfed agriculture receives only green water.

Sustainable Development Goal (SDG) target 6.4 aims to increase blue water efficiency and reduce blue water scarcity (UN, 2018; Unver et al., 2017; Vanham et al., 2018). It uses two indicators: 6.4.1 on water efficiency and 6.4.2 on water stress. The latter is defined as the ratio between water use and environmentally available water resources. Environmentally available water resources are available water resources minus environmental flows. Although both SDG indicators use water abstraction, also water consumption – as used in the WF - is relevant for both indicators (Vanham et al., 2018). Blue water stress assessments with this indicator are multiple (Gerten et al., 2020; Mekonnen and Hoekstra, 2016), as are water efficiency or water productivity assessments for crops, mostly by including blue and green water (Bastiaanssen and Steduto, 2017; Brauman et al., 2013; Karimi et al., 2013; Marston et al., 2020; Mekonnen and Hoekstra, 2014; Nouri et al., 2019). The water quantity sustainability scheme as proposed by Vanham and Leip (2020) builds on these two SDG indicators and addresses water consumption.

The scarcity-weighted WF has been developed by the LCA community, and is recommended in ISO document 14046 on water footprints (ISO, 2014). It is an LCA mid-point indicator, obtained by multiplying a WF with water stress, resulting in so-called water equivalents. Hoekstra (2016) first expressed concerns about the validity and physical meaning of this indicator. Although a general reply to his criticism was given (Pfister et al., 2017), his concerns remain valid, as the physical significance of this indicator has never been evaluated. Vanham and Leip (2020) recently observed that a consumer choice for an irrigated crop depends on which indicator is used – the blue WF, blue water stress or the scarcity-weighted WF. Main reason, as they pointed out, is because water use and water stress are only weakly correlated. In addition, Vanham (2020) found that for global irrigated maize, the scarcity-weighted WF partly recommends unsustainably produced maize over sustainably produced maize.

Here we provide a first detailed assessment of the consistency of scarcity-weighted WF scoring results with the two established indicators blue water stress and water efficiency, for the case of global irrigated wheat production. We choose irrigated wheat, as this crop has the largest blue WF as well as the largest unsustainable blue WF of all crops globally (Mekonnen and Hoekstra, 2020). We use the water quantity sustainability scheme by Vanham and Leip (2020) as base. Regarding water efficiency, we use two approaches. First, we use green and blue WF components on a global level with a globally set benchmark (Mekonnen and Hoekstra, 2014). Second, we use only the blue WF component, and account for different climatological zones as defined with an aridity index (Brauman et al., 2013; Cherlet et al., 2018; UNEP, 1992). We then identify benchmarks for each of these aridity zones.

## 2 Methodology

We use different spatially distributed global datasets of high quality. We use the blue and green WF of production and irrigated yield and production data of Mekonnen and Hoekstra (2011). These are global gridded data at a resolution of 5 by 5 arc minute (or 10 by 10 km at the equator). The global average blue and green WF of irrigated wheat are 926 respectively 679 m^3^/ton (average yield 3.3 ton/ha), with a total blue and green WF of 204 respectively 150 km^3^/year.

We use the spatially distributed blue water stress data of Mekonnen and Hoekstra (2016). This indicator puts blue WF amounts in relationship to local blue water availability. Blue water stress in Mekonnen and Hoekstra (2016) is computed as:

(1) $$\text{blue water stress}=\frac{\mathrm{blue}\;\mathrm{WF}}{\left(\mathrm{WA}-\mathrm{EFR}\right)}$$

With WA = total blue water availability, EFR = environmental flow requirements, and WA-EFR = environmentally available blue water resources.

Blue water stress values up to 1 are defined as “low water stress”. Values exceeding 1 indicate blue water stress.

Water use is a pressure indicator, whereas water stress is an impact indicator (Hoekstra and Wiedmann, 2014; Vanham et al., 2019). Pressure indicators quantify resource use and/or pollution, whereas impact indicators measure the impact caused by such pressures. LCA includes a life cycle inventory and life cycle impact assessment phase. In latter phase, it uses mid-point and end-point indicators to assess (environmental) impact. As mid-point indicator, the scarcity-weighted WF, also called water scarcity footprint, is used (Hoekstra, 2016; ISO, 2014). The scarcity-weighted WF (expressed in “*water-equivalent*”, “*H*_*2*_*O-equivalent*” or “*H*_*2*_*Oe*”) is obtained by multiplying the blue WF with water stress, with the latter referred to as “characterization factor”:

(2) scarcity weightedWF=blueWF∗blue water stress

To address water efficiency or water productivity, we use two benchmarks approaches. The first is a global benchmark based upon the blue plus green WF of wheat, which can be set at the 50th production percentile (1391 m^3^/ton) (Mekonnen and Hoekstra, 2014). Setting a global benchmark for blue plus green water for a certain crop has been conducted by many different authors (Mekonnen and Hoekstra, 2014; Rattalino Edreira et al., 2018; Zwart and Bastiaanssen, 2004). The second approach is benchmark amounts for only the blue WF, according to climate zones defined by an aridity index. Setting a global blue WF benchmark for irrigated wheat does not make sense, because a benchmark blue WF depends on the climate zone it is produced in (Brauman et al., 2013; Zhuo et al., 2016). All analyses are conducted as annual averages.

The aridity index is a numerical indicator of aridity based on long-term climatic water deficits and is calculated as the ratio of precipitation to potential evapotranspiration (P/PET) (Cherlet et al., 2018). It is a widely used measure of dryness of the climate at a given location. Six classes of aridity are generally classified: cold, hyper-arid, arid, semi-arid, dry subhumid and humid. Here we use the data presented in the most recent Atlas of Desertification (long time averages for 1981–2010) (JRC, 2018), based on Spinoni et al. (2015).

We compute for 248,654 grid cells whether irrigated wheat is produced sustainably or unsustainably within a grid cell. Sustainably produced wheat is produced under low water stress (value from 0 to 1) and in an efficient way (with a blue plus green Wf or blue WF upto a certain benchmark value). For unsustainably produced wheat, we distinguish three classes: 1) inefficient (a blue plus green WF or blue WF higher than the benchmark, but blue water stress up to 1); 2) water stress (a blue plus green WF or blue WF lower than the benchmark, but blue water stress exceeding 1) and 3) inefficient and water stress (a blue plus green WF or blue WF higher than the benchmark and blue water stress exceeding 1). We compute for each grid cell the scarcity-weighted WF and rank grid cells from lowest value (rank 1) to highest value (rank 248,654). We then compare this ranking with the results on sustainability.

## 3 Results

### 3.1 Evaluation by using a global benchmark for the blue plus green WF of wheat

**Fig. 1:**
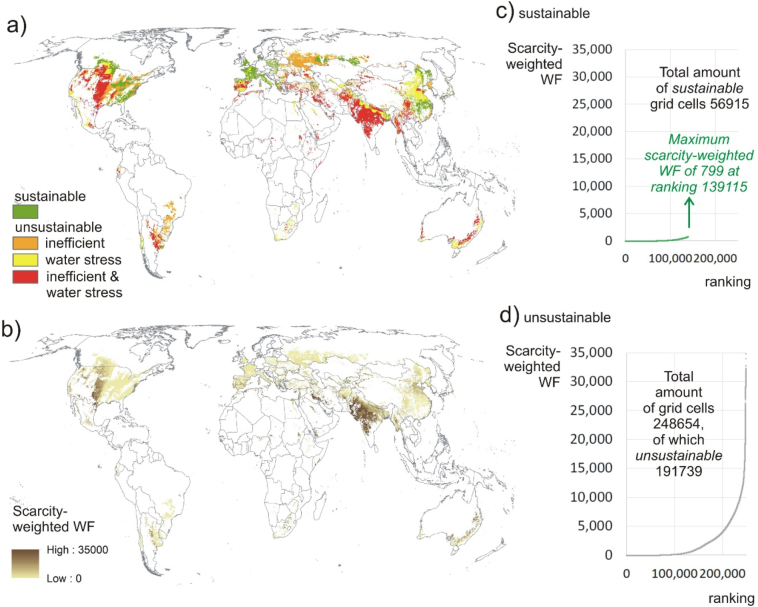
a) Map displaying the sustainability of the blue WF of irrigated wheat production, by using a global benchmark for the blue plus green WF of wheat; b) map displaying the scarcity-weighted WF of irrigated wheat, with related ranking of c) sustainable grid cells as well as d) unsustainable grid cells. (For interpretation of the references to colour in this figure legend, the reader is referred to the web version of this article.)

Fig. 1 displays a high spatial resolution global map depicting for a total of 248,654 grid cells the sustainability of the blue WF of irrigated wheat production, by using the global benchmark 1391 m^3^/ton for the blue plus green WF of wheat. In total, there are 56,915 sustainable grid cells and 191,739 unsustainable grid cells.

Fig. 1 also displays a map of the scarcity-weighted WF of irrigated wheat for each grid cell. Ranking latter amounts from low to high (1 to 248,654), results in a scarcity-weighted WF of 799 water equivalent being identified as the highest amount for all sustainable grid cells. This value has a ranking of 139,115. In total the 56,915 sustainable grid cells are ranked over a range of 1 to 139,115 (Fig. 1c). The 191,739 unsustainable grid cells are ranked over the whole range from 1 to 248,654. This thus means that up to the rank of 139,115, a substantial amount of unsustainable grid cells receive a better ranking than many sustainable grid cells.

**Fig. 2:**
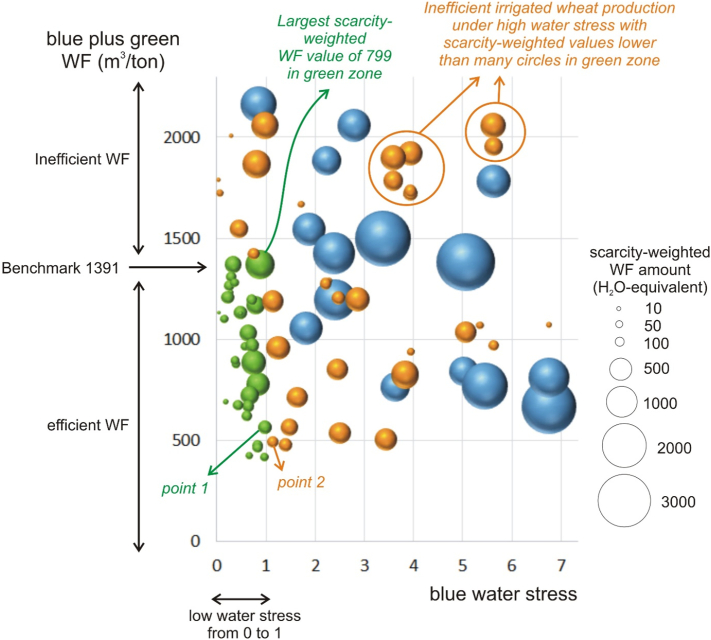
Water quantity sustainability scheme by Vanham and Leip (2020), displaying blue water stress (X-axis) and the blue plus green WF (Y-axis) for a sample of irrigated wheat grid cells. The relative size of each circle (grid cell) relates to the scarcity-weighted WF amount. Green circles indicate sustainable situations (low water stress and WF below the benchmark). Blue and orange circles indicate unsustainable situations. Orange circles identify lower scarcity-weighted WF amounts in the unsustainable zone as compared to the highest scarcity-weighted WF amount in the sustainable zone (799 water-equivalent). Blue circles identify higher scarcity-weighted WF amounts. (For interpretation of the references to colour in this figure legend, the reader is referred to the web version of this article.)

A sample of such grid cells is presented in the water quantity sustainability scheme by Vanham and Leip (2020), constituted of an X-axis displaying blue water stress and a Y-axis displaying the blue plus green WF (Fig. 2). The size of the circle of a grid cell relates to the amount of its scarcity-weighted WF. Green circles are in the sustainable zone, blue and orange points are in the unsustainable zones. Orange circles identify lower scarcity-weighted WF amounts in the unsustainable zone as compared to the highest scarcity-weighted WF amount in the sustainable zone (799 water equivalent).

A substantial amount of orange circles are observed under high water stress conditions as well as above the WF threshold value. These represent wheat production grid cells that have lower ranking than some of the green zone wheat production grid cells, when using the scarcity-weighted WF as sustainability indicator. The blue circles represent wheat production grid cells that are unsustainable and which are not ranked lower by the scarcity-weighted WF as compared to the locations in the green zone. In other words, the wheat of some farmers producing their crop in a low-impact and efficient manner, would be scored less sustainable as compared to some farmers producing in an unsustainable way. This is a conflicting information.

As an example, point 1 in Fig. 2 represents sustainably produced wheat in the green zone, with a water stress value of 0.98 (low water stress). With a green plus blue WF of 563 m^3^/ton (of which a blue WF of 152 m^3^/ton), it is efficiently produced (lower than the benchmark 1391 m^3^/ton). The scarcity-weighted WF amounts to 150 H_2_O-equivalent (0.98 multiplied with 152, Eq. (2)). Point 2 in Fig. 2 represents wheat produced under water stress (value 1.14), but efficiently (green plus blue WF of 491 m^3^/ton (of which blue 103 m^3^/ton)). Its scarcity-weighted WF amounts to 118 H_2_O-equivalent (1,14 multiplied with 103, Eq. (2)). Although unsustainably produced (it is orange coloured), its scarcity-weighted WF is thus lower and therefore better ranked compared to point 1, which represents sustainably produced wheat.

**Fig. 3:**
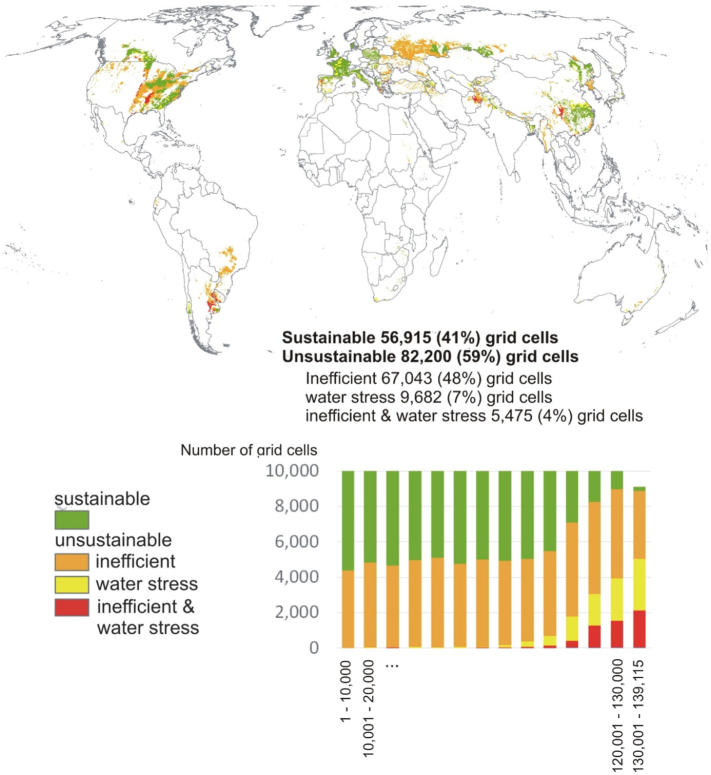
Sustainability of irrigated wheat grid cells ranked according to the scarcity-weighted WF from 1 to 139,115, with (bottom figure) differentiation between 14 ranking intervals (steps of 10,000 grid cells). The ranking value 139,115 represents the highest scarcity-weighted WF (799 water equivalent) that is sustainable.

Within the ranking 1 to 139,115, 56,915 (41%) grid cells are sustainable and 82,200 (59%) are unsustainable (Fig. 3). All three classes of unsustainable wheat production are represented throughout the range: inefficient (67,043 grid cells or 48% of range), water stress (9862 grid cells or 7%) as well as inefficient and water stress (5475 grid cells or 4%). When differentiating this ranking between 14 intervals of 10,000 grid cells, high percentages of unsustainable grid cells are observed in all intervals (Fig. 3). This means that spread over the entire range, unsustainable wheat production has lower (better) ranking scores than sustainable grid cells.

### 3.2 Evaluation by using different benchmark amounts for the blue WF

**Fig. 4:**
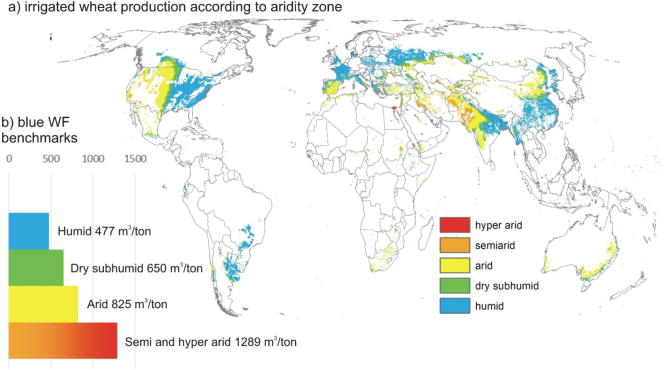
a) Irrigated wheat production in different aridity zones: humid, dry subhumid, arid, semiarid and hyper arid; b) blue WF benchmarks (m^3^/ton) according to aridity zones (semi and hyper arid zones are converged to one zone). (For interpretation of the references to colour in this figure legend, the reader is referred to the web version of this article.)

In a second approach to address water efficiency, we use only the blue WF component for defining a benchmark, and account for different climatological zones based on an aridity index (Fig. 4a). For each aridity zone we compute a benchmark, set at the 50th production percentile (Fig. 4b): 477 m^3^/ton for the humid zone, 650 m^3^/ton for the dry subhumid zone, 825 m^3^/ton for the arid zone and 1289 m^3^/ton for the semi and hyper arid zones combined. Latter are merged as they account relatively for much fewer grid cells.

**Fig. 5:**
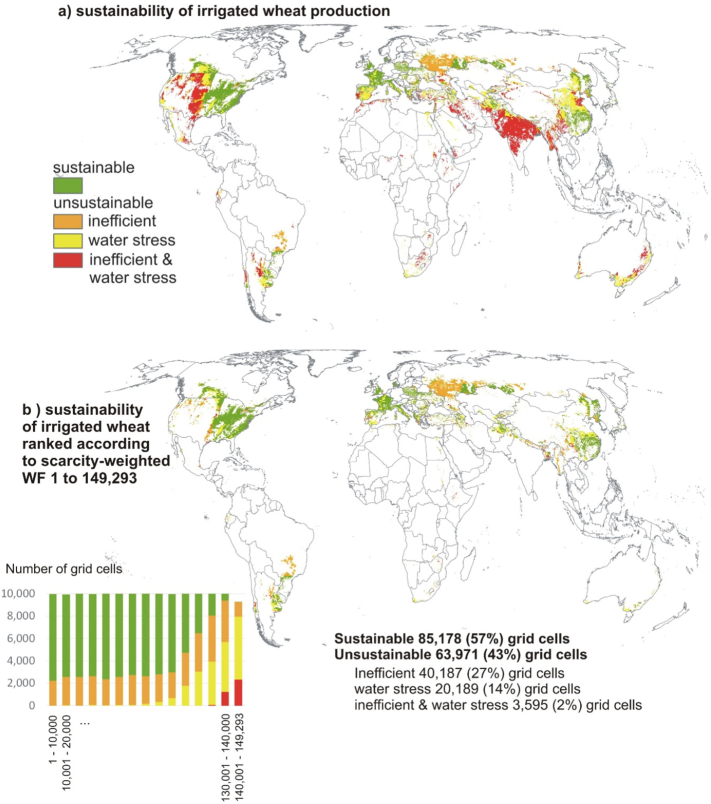
a) Map displaying the sustainability of irrigated wheat production, using different blue WF benchmarks according to aridity zones; b) sustainability of irrigated wheat grid cells ranked according to the scarcity-weighted WF from 1 to 149,293, with differentiation between 15 ranking intervals (steps of 10,000 grid cells). (For interpretation of the references to colour in this figure legend, the reader is referred to the web version of this article.)

Based on these blue WF benchmarks as well as blue water stress, the overall sustainability of irrigated wheat is then quantified, as displayed in Fig. 5. Fig. 5a is very similar to Fig. 1a, but there are some differences, which is logical as the WF benchmark settings are different. A scarcity-weighted WF of 1188 water equivalent is identified as the highest amount for all sustainable grid cells, with a ranking of 149,293 (Fig. 5b). Within the ranking range from 1 to 149,293, 85,178 (57%) grid cells are sustainable and 63,971 (43%) are unsustainable. For the latter, all three classes of unsustainable wheat production are represented throughout the range: inefficient (40,187 grid cells), water stress (20,189 grid cells) as well as inefficient and water stress (3595 grid cells). Again, significant percentages of unsustainable grid cells are represented in 15 ranking intervals of 10,000 grid cells.

## 4 Discussion

Our results show that a significant amount of grid cells where wheat is produced in a sustainable way receive higher scarcity-weighted WF amounts (they are ranked worse) as compared to a significant amount of grid cells where wheat is produced in an unsustainable way. Many farmers producing wheat sustainably thus would be ranked worse than many farmers producing wheat unsustainably, when applying the scarcity-weighted WF as indicator.

We have observed this for the ranking range from 1 to the sustainable grid cell with the highest scarcity-weighted WF amount, i.e. 1 to 139,115 (scarcity-weighted WF of 799 water equivalent) when applying a global blue plus green WF benchmark and 1 to 149,293 (scarcity-weighted WF of 1188 water equivalent) when applying different blue WF benchmarks. Unsustainably produced wheat within these ranges includes all three classes of unsustainable wheat production: inefficient, water stress as well as inefficient and water stress. Above these highest ranked sustainable grid cells, the meaningfulness of the ranking range of only unsustainably produced wheat is unclear. Why a product has a lower or higher score than another one, cannot be accounted for.

Hoekstra (2016) already pointed out that the formula to calculate a scarcity-weighted WF implies the ratio between two times the blue WF and environmentally available blue water (Eq. (3), obtained by combining Eqs. (1), (2)), which has no logic. Combined with the fact that the blue WF and blue WS are only weakly correlated (Vanham and Leip, 2020), this formula to calculate the scarcity-weighted WF provides unreliable scoring results as observed in our paper.

(3) $$\text{scarcity weighted}\;\mathrm{WF}=\mathrm{blue}\;\mathrm{WF}*\mathrm{blue water stress}=\frac{\mathrm{blue}\;\mathrm{WF}*\mathrm{blue}\;\mathrm{WF}}{\left(\mathrm{WA}-\mathrm{EFR}\right)}$$

To address water efficiency, we use a global green plus blue WF benchmark as well as different blue WF benchmarks according to aridity zones. The resulting amounts of grid cells defined as sustainable differ for the two approaches, which is logical. However, the conclusions on the random scoring for the scarcity-weighted WF are in line. Setting a blue WF benchmark is sensitive to climatological conditions, and detailed considerations of more specific aridity and climate zones will result in better blue WF benchmark estimations. Nevertheless, the green plus blue global WF benchmark methodology, in line with water productivity assessments, is the recommended procedure. Vanham and Leip (2020) use latter procedure.

The scarcity-weighted WF and scarcity-weighted water use have been applied in a large number of LCA-based studies. This indicator is used in publications in high-profile journals such as Science (Poore and Nemecek, 2018) or PNAS (Clark et al., 2019), where the authors use it among other environmental indicators to evaluate the sustainability of food products. Our assessment puts the results and recommendations of studies that use this specific indicator into question, as this indicator does not result in a scoring representing sustainable water use.

Sustainable food system policies should not use this indicator, as it is unfair for all actors along the supply chain, from agricultural producers to consumers. Policies for incentivizing farmers to produce in a water-sustainable way or for stimulating consumers to buy water-sustainable food products would be contraproductive in actually delivering on reaching SDG target 6.4, when using this indicator. The Farm to Fork Strategy of the European Green Deal is a policy that aims at making the food system more environmentally sustainable (EC, 2020a; EC, 2020b; Vanham and Leip, 2020). It includes the implementation of a sustainable food labelling framework, to empower consumers to make sustainable food choices. This and other food labelling initiatives should not use the scarcity-weighted WF as indicator.

Using the two indicators water efficiency and water stress separately in a complementary way is needed to measure progress to and to deliver on SDG target 6.4. Achieving WF or water productivity benchmarks is in line with the sustainable intensification of food production (Springmann et al., 2018; Willett et al., 2019) and the achievement of low water stress will deliver on maintaining environmental flows, essential for biodiversity and multiple ecosystem services (Falkenmark et al., 2019; Vanham et al., 2018). The whole SDG framework contains 230 individual indicators to monitor the 17 goals and 169 targets. There are thus more indicators than targets. As shown in this paper, attempts to combine water use and water stress in one scarcity-weighted water use indicator provide meaningless numbers. Certain indicators need to be kept separate. The scheme proposed by Vanham and Leip (2020) uses the two indicators related to target 6.4 separately.

## 5 Conclusions

This study is the first detailed assessment to test whether the scarcity-weighted WF, also referred to as water scarcity footprint, grasps sustainable water use in a physically meaningful way. We evaluated for irrigated wheat production, in high spatial resolution (248,654 grid cells) covering the globe, whether the ranking resulting from scarcity-weighted WF values is in line with the two widely accepted indicators water efficiency and blue water stress. We find that the scarcity-weighted WF provides inconsistent scoring results with respect to these two indicators. A large proportion of unsustainably produced irrigated wheat is ranked better (lower values on the ranking range) as compared to a large proportion of sustainably produced irrigated wheat. Better ranked unsustainable wheat includes wheat produced under blue water stress, inefficiently produced wheat as well as wheat both produced under blue water stress and inefficiently. In other words, wheat from farmers producing in a sustainable manner is often scored worse (higher scarcity-weighted WF amounts) than wheat from farmers producing in an unsustainable manner.

Our assessment clearly shows that multiplying a WF with a water stress indicator results in an indicator with unclear physical meaning, not properly providing better (lower) scores for sustainably produced wheat over unsustainably produced wheat. The scarcity-weighted WF is therefore unfair for all actors along the supply chain, from agricultural producers to final consumers. Producers are not valued or rewarded for their efforts to sustainably produce wheat. Consumers are provided with false product information when a sustainability label includes the scarcity-weighted WF. The European Farm to Fork Strategy includes the implementation of such a sustainable food labelling framework - currently still under development -, to empower consumers to make sustainable food choices.

LCA assessments may use particular other water stress indicators as the one used here (FAO, 2019; Hoekstra, 2016), but that is not relevant with respect to our evaluation. Multiplying a WF with a water stress indicator provides meaningless amounts. In addition, using multiple scarcity-weighted WF amounts for different products, to achieve a scarcity-weighted WF amount for a diet, will also result in unreliable values. A scarcity-weighted WF is expressed in water equivalents (or H_2_Oe), which are not real water volumes and should not be used or communicated as such.

The scarcity-weighted WF and scarcity-weighted water use are recommended in different documents (ISO, 2014) and have been widely applied in LCA-based studies, including in high-profile journals. Our assessment is the first detailed case study assessment to put the results of those studies and resulting decision-making recommendations into question, as they may hamper reaching SDG target 6.4.

To properly evaluate the sustainability of water use and measure progress towards reducing water stress, the two indicators water stress and water efficiency are to be used separately, in a complementary manner. Measuring progress towards SDG target 6.4 by means of these two indicators is the correct procedure, as put in place in the current SDG framework.

## CRediT authorship contribution statement

**Davy Vanham:** Conceptualization, Data analysis, Visualization, Writing- Original draft preparation

**Mesfin M Mekonnen**: Data provision, Writing- Reviewing and Editing.

## Declaration of competing interest

The authors declare that they have no known competing financial interests or personal relationships that could have appeared to influence the work reported in this paper.
